# AGE-ASSOCIATED DECLINE IN TISSUE CIRCADIAN CLOCK FUNCTION

**DOI:** 10.1093/geroni/igad104.1679

**Published:** 2023-12-21

**Authors:** Karyn Esser

**Affiliations:** University of Florida, Gainesville, Florida, United States

## Abstract

Cellular circadian clocks direct a daily transcriptional program that supports homeostasis and resilience. Emerging evidence has demonstrated age-associated changes in circadian functions. To define age-dependent changes at the systems level, we profile the circadian transcriptome in the hypothalamus, lung, heart, kidney, skeletal muscle, and adrenal gland in three age groups. We find age-dependent and tissue-specific clock output changes but these changes exhibit tissue specific patterns. In general, aging reduces the number of rhythmically expressed genes (REGs), indicative of weakened circadian control. These REGs are enriched for the hallmarks of aging, adding another dimension to our understanding of aging. We determined there were tissue specific changes in the phase distribution of REGs with the hypothalamus and skeletal muscle tissues of the old mice showing a large reduction with REG peak expression at only one phase of the day. Analyzing all expressed genes within a tissue for differences at four distinct times of day identified unique clusters of differentially expressed genes (DEGs). These outcomes identify non-circadian but temporally distinct age associated changes in gene expression. This analysis extends the landscape for understanding aging and highlights the impact of aging on circadian clock function and temporal changes in gene expression.

